# Supplementary material for: A Global Screen for Assembly State Changes of the Mitotic Proteome by SEC-SWATH-MS
Source: Cell Syst. 2020 Feb 26;10(2):133–155.e6. doi: 10.1016/j.cels.2020.01.001 (PMC7042714; doi:10.1016/j.cels.2020.01.001)

Q14155 | ARHG7_HUMAN | ARHGEF7 COOL1 KIAA0142 P85SPR PAK3BP PIXB Nbla10314
Monomer MW [kDa]: 90.012 Monomer expected elution fraction: 41

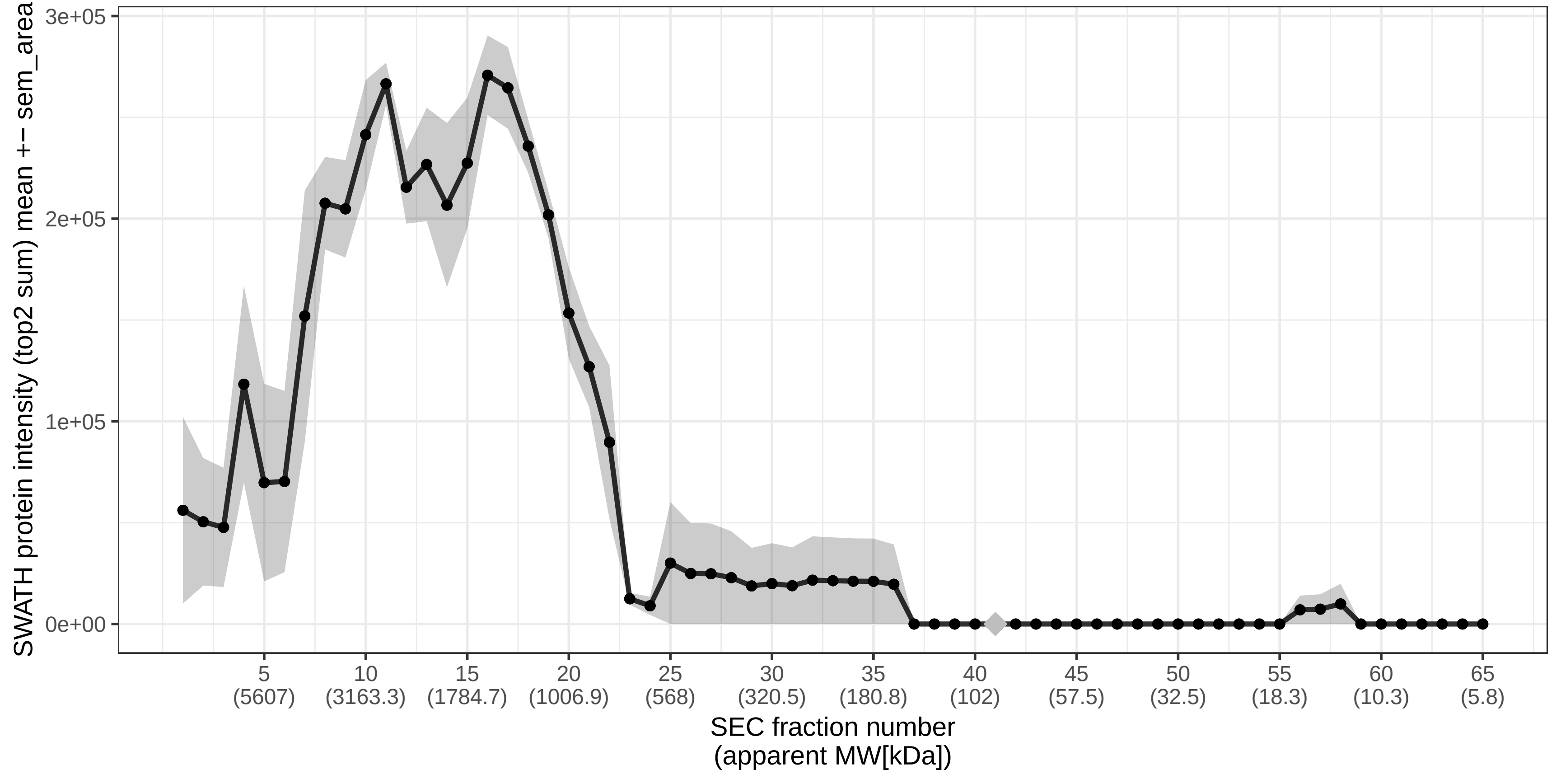

Supplement: Data S1. SEC-SWATH-MS Protein Chromatograms, Related to Figure 1 [file mmc6.zip › SECchrom_Q14155_ARHG7_HUMAN_ARHGEF7_COOL1_KIAA0142_P85SPR_P.pdf]
